# Effects of the 2018 Camp Fire on birth outcomes in non-human primates: Case-control study

**DOI:** 10.1016/j.reprotox.2021.08.005

**Published:** 2021-08-25

**Authors:** Bryn E. Willson, Nancy A. Gee, Neil H. Willits, Lijuan Li, Qi Zhang, Kent E. Pinkerton, Bill L. Lasley

**Affiliations:** a UC Davis Health, University of California, Davis, CA, Davis, 95616, United States; b Center for Health and the Environment, University of California, Davis, CA, Davis, 95616, United States; c California National Primate Research Center, University of California, Davis, CA, Davis, 95616, United States; d Department of Biostatistics, University of California, Davis, CA, Davis, 95616, United States; e Department of Environmental Toxicology, University of California, Davis, CA, Davis, 95616, United States

**Keywords:** Wildfire, Smoke, Air-quality, Climate, Particulate, Primate, Phthalate, Miscarriage

## Abstract

The November 2018 Camp Fire, a devastating wildfire in Northern California, occurred during the peak of breeding season for field monkeys at the California National Primate Research Center (CNPRC). Effects of environmental stressors, such as wildfires, on birth outcomes in primates, and in humans, are poorly understood. Additionally, wildfires are of growing concern due to their increasing frequency and severity. The objective was to examine the impact of wildfire smoke on fertility, timing of birth, and pregnancy loss for field monkeys.

A unique case-control study to investigate birth outcomes in rhesus macaques (*Macaca mulatta*) was conducted at the CNPRC. All females in the study were maintained in outdoor fields during a period of elevated ambient wildfire smoke from November 8–22, 2018. In addition to ambient air quality evaluations, the effects on fertility, timing to birth, and pregnancy loss were documented. Archival records of approximately 5,000 conceptions from the previous nine years served as control data.

During the Camp Fire, ambient fine particulate (PM _2.5_) levels exceeded the 24 -h National Ambient Air Quality Standard (35 μg/m 3) of the United States Environmental Protection Agency, reaching levels as high as 185 μg/m 3. A statistically significant association was observed between birth loss and the 2018–2019 CNPRC breeding season. As this wildfire event occurred during various stages of early pregnancy, an association can be inferred between early gestational exposure and increased risk of pregnancy loss.

## 1. Introduction

In November 2018, the Camp Fire wildfire became the deadliest wildfire in California history documented by the California Department of Forestry and Fire Protection. The poor air quality arising from the wide-spread dissemination of wildfire smoke provided the opportunity to study its effects on birth outcomes in non-human primates residing outdoors at the California National Primate Research Center (CNPRC) in Davis, CA, a city approximately 160 km away from the origin of the Camp Fire disaster.

Wildfire air pollution is composed primarily of organic and inorganic carbonaceous particulate matter (PM; [1]). One of the ways of quantifying wildfire air pollution is by measuring ambient levels of fine particulate matter (PM_2.5_). PM_2.5_ consists of particles that are ≤2.5 μm in diameter. Elevated PM_2.5_ levels have been described as the fifth leading risk factor contributing to global mortality [2]. A landmark study examining over 800,000 pregnancies during the 2003 wildfires in Southern California showed a decrease in neonatal birth weight for all women, irrespective of the exposure trimester [3]. Relatedly, a 2015 meta-analysis showed associations between elevated PM_2.5_ levels, increased risk of preterm birth, and decreased birth weight [4].

The aims of the present study were to examine the effects of a real- world exposure to one of the most serious and devastating wildfires in California history on rhesus macaque (*Macaca mulatta*) fertility, timing to birth, and pregnancy loss. The rhesus macaque is a logical non-human primate model for humans based on the length of gestation, type of placentation, and endocrine foundation of pregnancy. Thus, in the present study, rhesus macaques housed in outside enclosures at the CNPRC and exposed to a wide range of ambient environmental factors, even during their 24-week gestation period, were monitored to investigate the reproductive and developmental impacts of transported wildfire smoke.

The Camp Fire event occurred in Paradise, CA in November 2018, near the peak of the natural breeding season in the CNPRC’s outdoor field enclosures that housed over 500 mature female rhesus macaques. During the exposure period, all stages of gestation were represented in the study population. This consistent exposure across gestation permitted the exploration of the temporal relationship between wildfire smoke exposure and adverse pregnancy outcomes. We hypothesized adverse effects would cluster around a more sensitive period, early in gestation, and provide insights into the possible mechanism(s) by which air pollution alters higher-primate fetal development.

## 2. Methods

### 2.1. Study cohort and biological endpoints

Rhesus macaques breed in seasons, with conception occurring in the fall and winter months and delivery in the spring and summer months. The average gestation time of the rhesus macaque is 166.5 ±10 days [5]. The present case-control study was conducted in 66 reproductive-age female rhesus macaques residing outdoors at the CNRPC.

All outdoor CNRPC monkeys were exposed to ambient wildfire smoke arising from the Camp Fire wildfire that burned from November 8–22, 2018. Though the epicenter of the fire was approximately 160 km (100 miles) from the CNRPC, the resulting smoke decreased air quality to such a degree that it led to the unprecedented cessation of classes and coursework at the University of California, Davis campus for a period of two weeks.

The entire breeding colony, consisting of more than 500 monkeys, underwent routine management surveillance for conception and birth outcomes. Sixty-six females were selected at random, from various outdoor cages, during routine field examinations on December 4, 6, and 11, 2018, when air quality had returned to baseline levels (daily PM_2.5_ typically < 10 μg/m^−3^). These animals represented the 2018–2019 Camp Fire cohort. Handlers were blinded as to which primates would be followed for pregnancy outcomes, and the sample size was determined according to the limitations of funding available for primate handling and pregnancy assays. Inclusion criteria included female primates of reproductive age, residing in outdoor colonies. Exclusion criteria included primates that had received any form of contraception over the prior year.

### 2.2. Conception, live birth, and maternal demographic data

Total conceptions, conception rates, live births, and live birth rate per conception were determined for the cohort, along with demographic information regarding maternal age, gravidity (number of times pregnant), and parity (number of times given live birth). Pregnancy data were analyzed from nine preceding breeding years to act as a control for comparisons to the Camp Fire cohort.

### 2.3. Pregnancy status and chorionic gonadotropin levels in the camp fire cohort

Palpation of the abdomen by professional primate handlers was used to assess pregnancy status, and blood (serum) draws were completed to assess the presence of macaque chorionic gonadotropin (mCG), a marker of early pregnancy (gestational days 14–28). Macaque chorionic gonadotropin is similar to beta human chorionic gonadotropin (βhCG) in that it elevates in a predictable fashion in early pregnancy. However, mCG differs from βhCG in that the former is only measurable in blood serum from days 14–28 of gestation [6].

All pregnancies used in calculations (n = 54) were confirmed by palpation alone or palpation as well as mCG (n = 11). A previously validated assay for mCG was used [6]. The timing and frequency of conceptions by month were also determined. The Actual Conception Date (ACD) was calculated by taking the precise date the primate delivered a live born and subtracting 165 days, the average length of a macaque gestation (ACD delivery date – 165 days). When necessary, such as in the case of no live birth, the ACD was determined by the detection of mCG (n = 11) and/or by examination of the products of conception by experienced primate handlers. The ACD was then recorded for each of the conceptions that occurred during the 2018–2019 breeding season.

### 2.4. Population exposure: daily particulate matter

Daily PM exposure monitoring was simultaneously conducted on the University of California, Davis (UCD) campus at a dedicated air quality monitoring site operated by the California Air Resources Board (CARB). CARB-validated hourly measurements of ambient PM_2.5_ were provided using a PM_2.5_ Beta Attenuation Mass Monitor. The CARB site is three kilometers away from the CNPRC outdoor breeding colonies and located on Campbell Road, in Yolo County, in the Sacramento Valley Air Basin (Latitude 38.53455, Longitude −121.77340); therefore, it is representative of the ambient air to which the outdoor primates were exposed.

### 2.5. Population exposure: wildfire smoke chemical composition

To evaluate PM contributed by wildfire smoke as well as other sources (e.g., vehicle emissions and atmospheric chemical reactions), real-time aerosol chemistry measurements were made using a high- resolution time-of-flight aerosol mass spectrometer (HR-AMS) at the UCD campus during and after the Camp Fire event. HR-AMS enables the measurement of non-refractory constituents (i.e., chemical species that flash evaporate at ~ 600 °;C – the HR-AMS vaporizer temperature) in submicrometer particles (PM_1_; [7]). These constituents include but are not limited to sulfate, nitrate, ammonium, chloride, and organic matter (e.g., plant and animal biomass material), which typically account for a major mass fraction of ambient PM_2.5_. Common refractory PM constituents like black carbon, dust, and crustal material which evaporate at much higher temperatures are not measured by HR-AMS.

### 2.6. Statistical analysis of smoke composition

Statistical analysis of the aerosol mass spectral data was performed using Positive Matrix Factorization (PMF) to quantitatively determine the contributions of the different PM sources during the Camp Fire. The mass spectra of the different organic PM types in the air samples collected for the present study were compared with the spectra of known organic PM types to evaluate chemical similarities and differences.

### 2.7. Statistical analysis of pregnancy data

A non-parametric regression additive model was utilized in order to assess pregnancy outcomes during 2018 2019 breeding season for the Camp Fire cohort. Adjustments were taken into consideration for maternal age and parity status. Additionally, the Odds Ratio (OR) and relative risk (RR) were calculated to assess the odds of pregnancy loss in the primates exposed to wildfire smoke during the Camp Fire versus primates that conceived during the nine preceding, relatively clean-air years.

## 3. Results

### 3.1. Conception, live birth, and maternal mCG data

Total conceptions, conception rates, live births, and live birth rate/conception are shown in Table 1 for each breading season cohort beginning with 2009 2010 and ending with the 2018 2019 Camp Fire- Exposed Cohort. The Camp Fire-Exposed Cohort included the pregnancies that were exposed to the high levels of fine particulates resulting from the Camp Fire wildfire event. Results indicated that while the total number of conceptions was artificially low, due to our selection of a limited number of animals for our experimental Camp Fire cohort (n = 66), live birth/total conceptions was the lowest at 82 % compared to the 9 preceding control breeding seasons (Table 1).

**Fig. 1.**
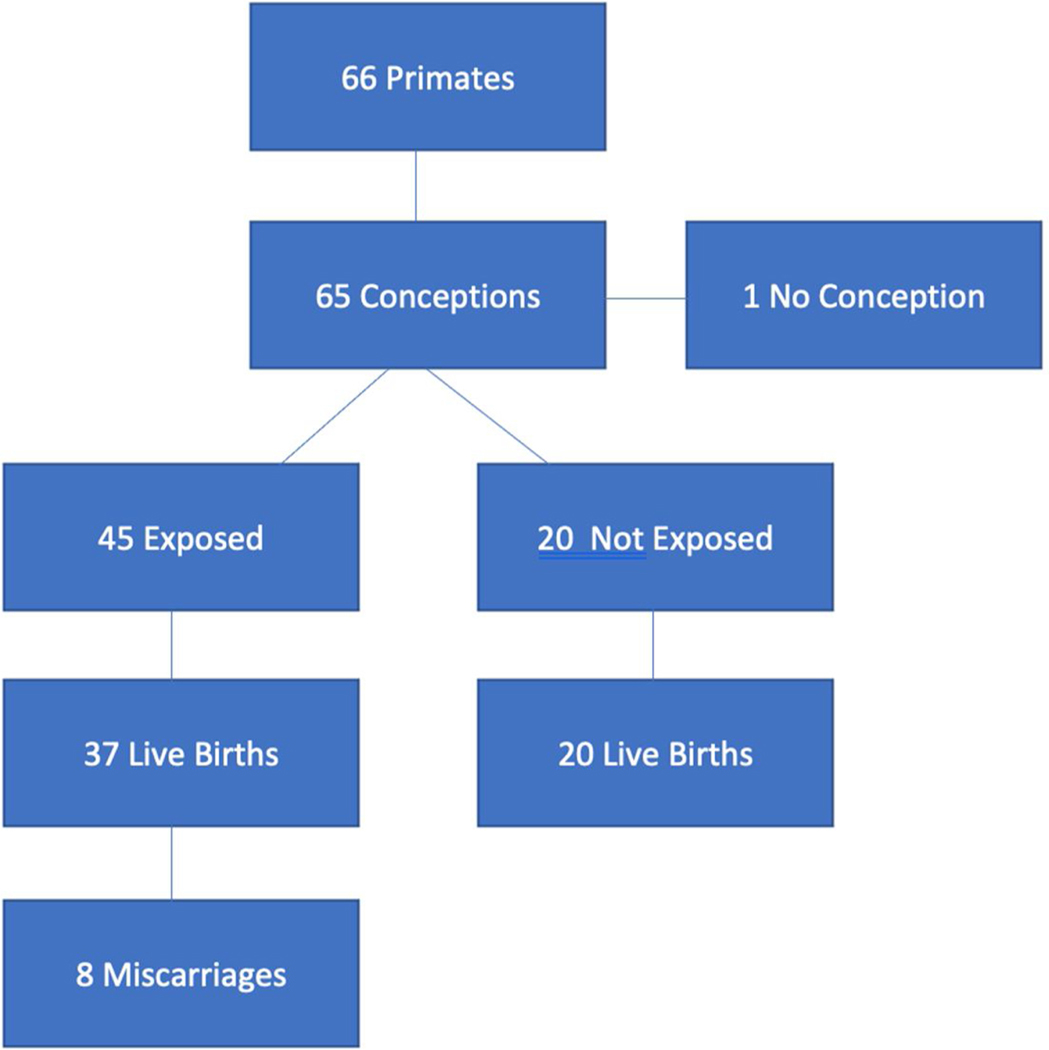
Flow chart of Camp Fire cohort birth outcomes after exposure to wildfire smoke during pregnancy.

Among the complete Camp Fire cohort selected for study (n = 66), 65/66 females conceived during the 2018–2019 breeding season. Of the 65 females that conceived, 45 were exposed to the elevated Camp Fire PM_2.5_ levels during pregnancy (Camp Fire-Exposed Cohort in Table 1), and 20 conceived shortly after air quality levels returned to normal (Fig. 1). Of the 45 females exposed during pregnancy, 37 went on to have live births and eight miscarried (defined as no live infant born after confirmation of pregnancy via palpation, presence of serum mCG, or delivery of products of conception; Fig. 1). Of the 20 conceptions not exposed to elevated levels of fine particulates, all 20 went on to have live births. The rate of live births among the non-exposed cohort is artificially high and is influenced by the overall small number of animals. This is best demonstrated by the average live birth rate from the 2009–2018 control group that ranged from 86 % to 93 % (Table 1).

Individual animal pregnancy confirmation and outcome data for the complete campfire cohort (n = 66) are shown in Table 2. Pregnancies were confirmed by palpation alone in 33/65 (51 %) of the cohort, palpation and mCG in 7/65 (11 %) of the cohort, mCG alone in 4/65 (6 %) of the cohort, and delivery of products of conception alone in 21/65 (32 %) of the cohort.

**Fig. 2.**
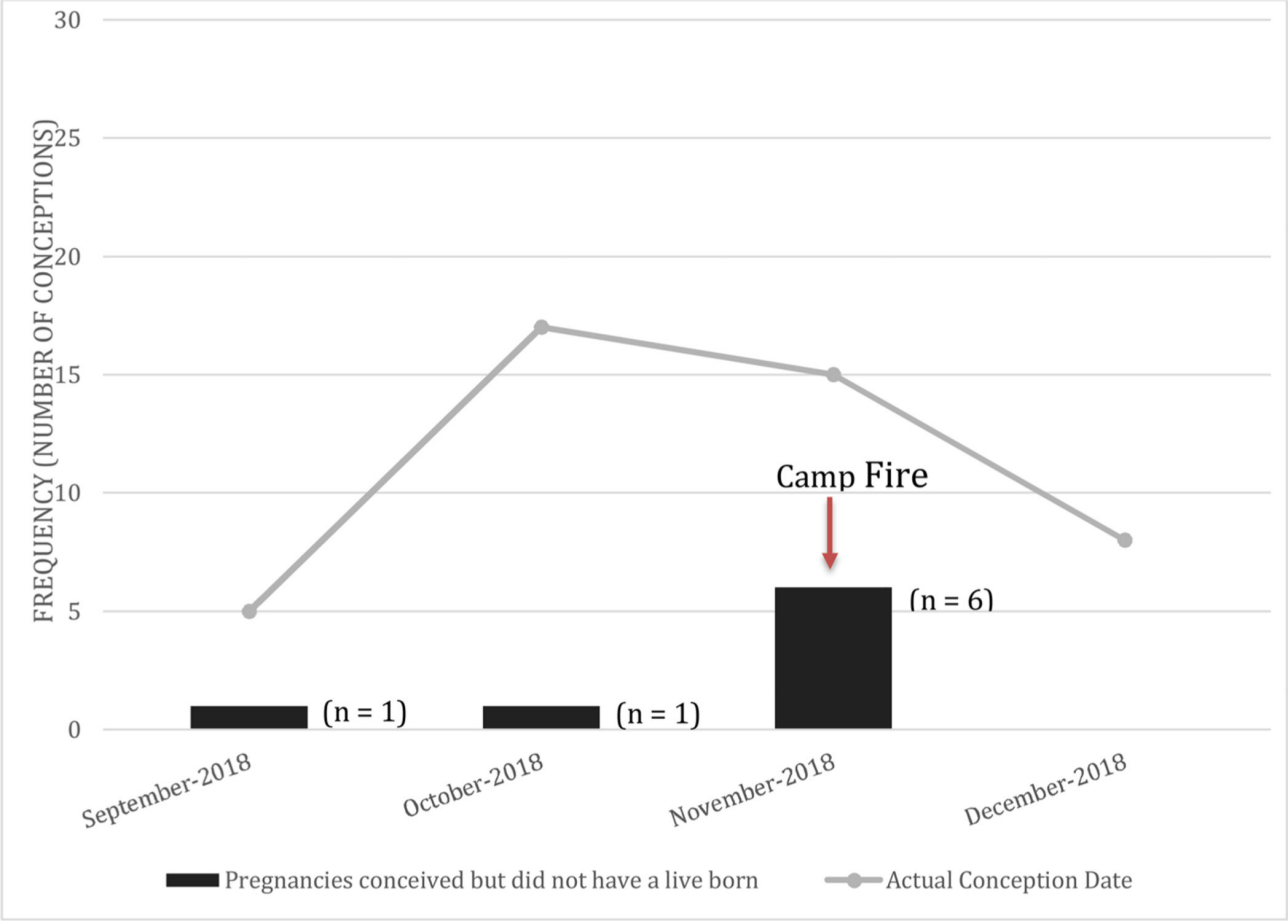
Timeline of conceptions during 2018–2019 breeding season. The frequency of total conceived pregnancies is shown for each month as it relates to the proportion that did not go on to live births.

Evaluation of the timing and frequency of conceptions by month (Fig. 2) demonstrated that most primates conceived between October and December 2018. Pregnancy loss was defined as no live birth after confirmation of pregnancy or delivery of products of conception, were tracked and noted to be clustered during the month of November 2018, the same month of the Camp Fire wildfire (n = 6; Fig. 2).

**Fig. 3.**
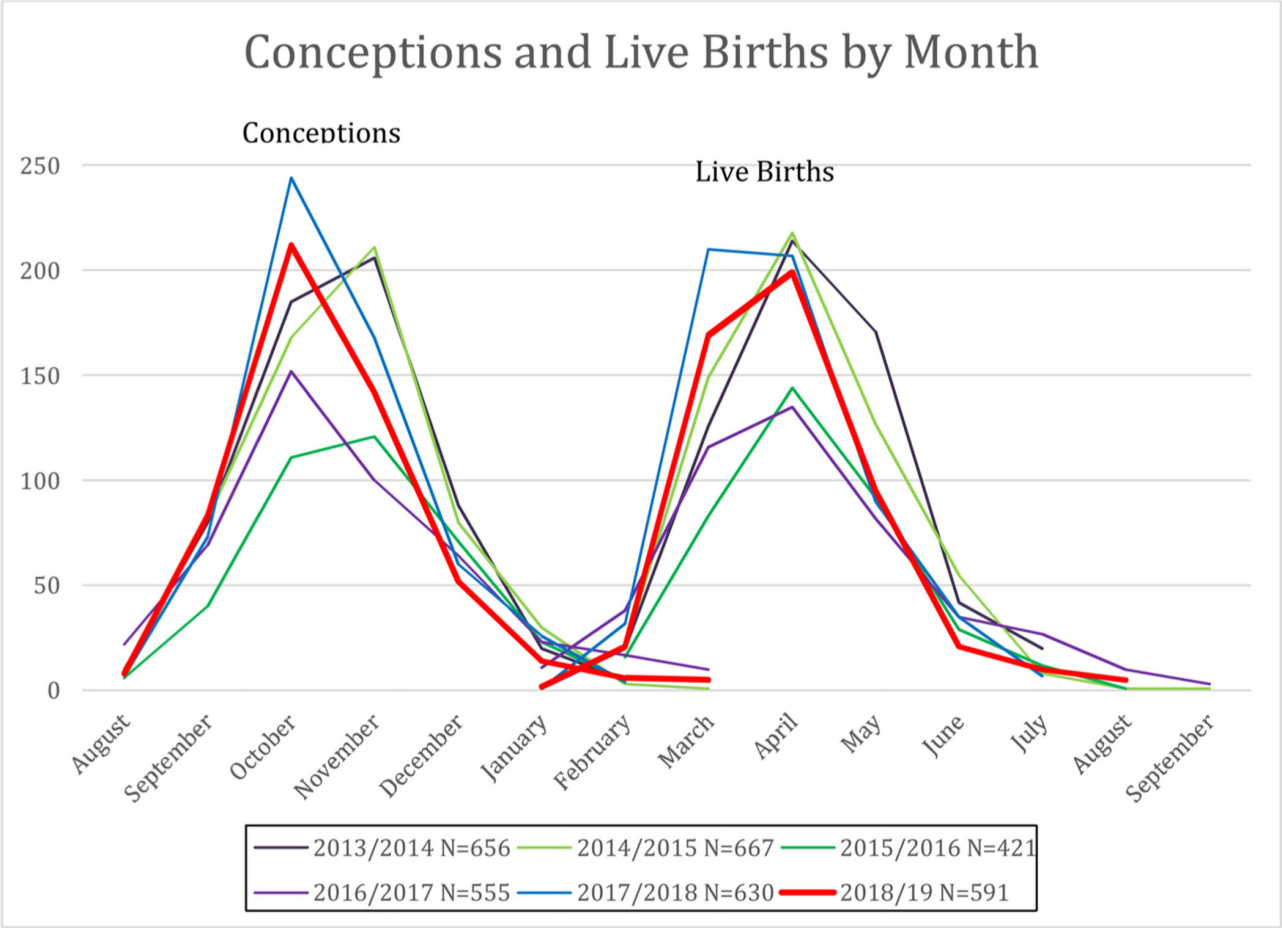
No change in seasonality or timeline of conception to live births from 2013–2019 for California National Primate Research Center outdoor primates.

Notably, the seasonality of the breeding season was not changed by the Camp Fire in terms of timing and duration of gestation, indicating little if any effect on time to conception and length of gestation (Fig. 3).

Additionally, comparisons of the proportions of pregnancy loss and live births as fractions of the total number of wildfire-smoke-exposed pregnancies in the Camp Fire cohort, and cumulative pregnancies (over 9 seasons) in the control cohort, showed that the percentage of pregnancy loss in the former was nearly double that of the latter (Table 3).

Table 3 compares conceptions from the 2018–2019 cohort exposed to Camp Fire wildfire smoke during various stages of pregnancy (n = 45) and all conceptions from the nine preceding breeding seasons (n = 5680) when the air was relatively clean. Pregnancy loss is defined as no live birth after confirmation of pregnancy from palpation, mCG assay, or delivery of products of conception.

**Fig. 4.**
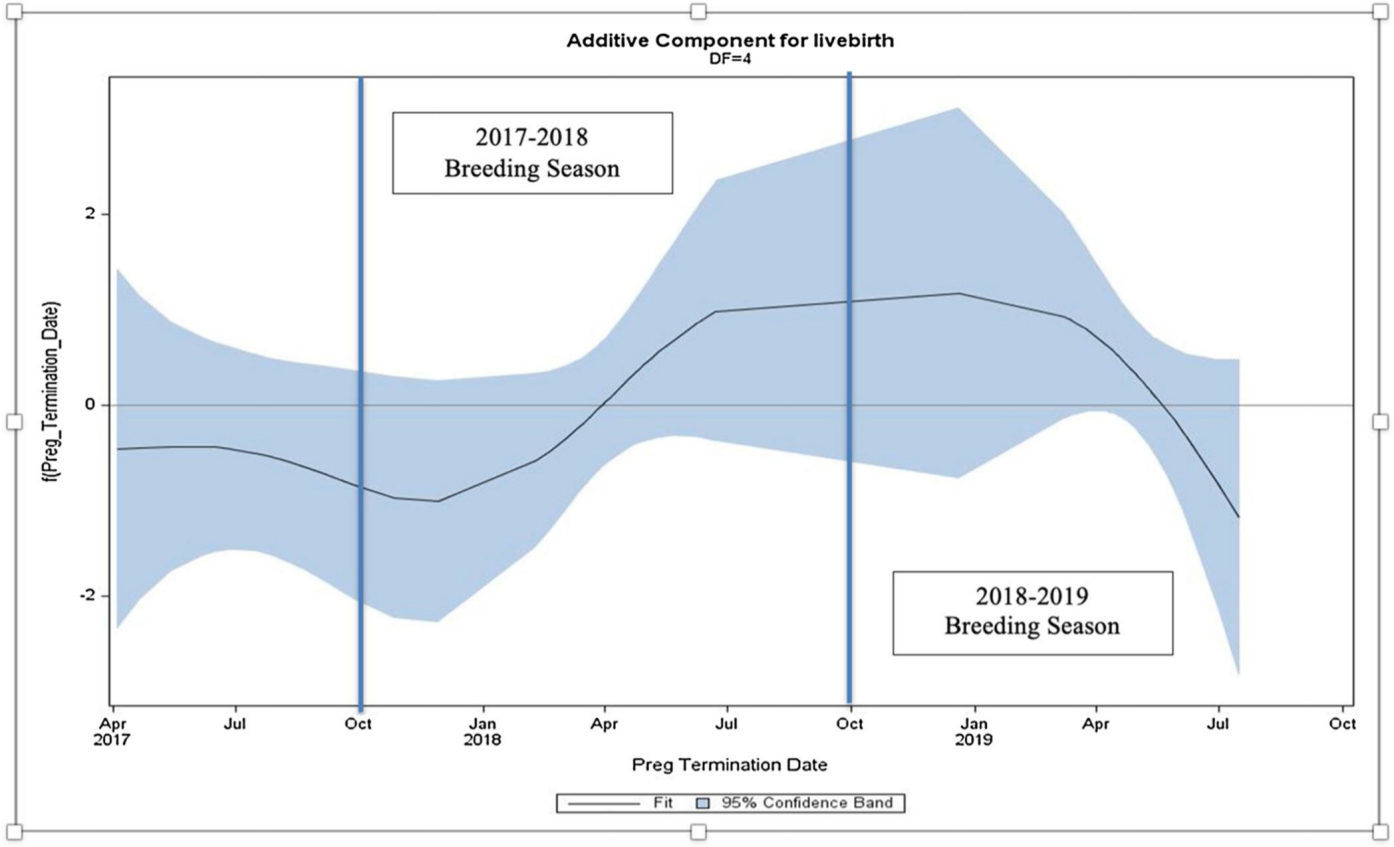
Additive model demonstrates a statistically significant trend toward pregnancy loss for the 2018–2019 breeding season (*p* = 0.035; when controlling for maternal age/parity status, *p* = 0.045).

Using an additive model adjusted for maternal age and parity for the 2018–2019 Camp Fire cohort, a statistically significant trend towards pregnancy loss was demonstrated by a logarithmic downward slope for the 2018–2019 breeding season (*p <* 0.045; Fig. 4). This figure depicts the preceding breeding season 2017–2018 with an upward slope or trend toward live birth for comparison (Fig. 4).

The OR calculated to assess the odds of pregnancy loss in the primates exposed to wildfire smoke during the Camp Fire versus primates that conceived during the nine preceding, relatively clean-air years was 1.969 (95 % confidence interval (CI) = 0.9124–4.2491; *p* = 0.0843). The RR was 1.7968 (95 % CI = 0.9538–3.3846; *p* = 0.0697; Table 3). The OR and RR although not statistically significant, given the small data set, the proximity to significance is important to note.

### 3.2. Daily PM concentrations

**Fig. 5.**
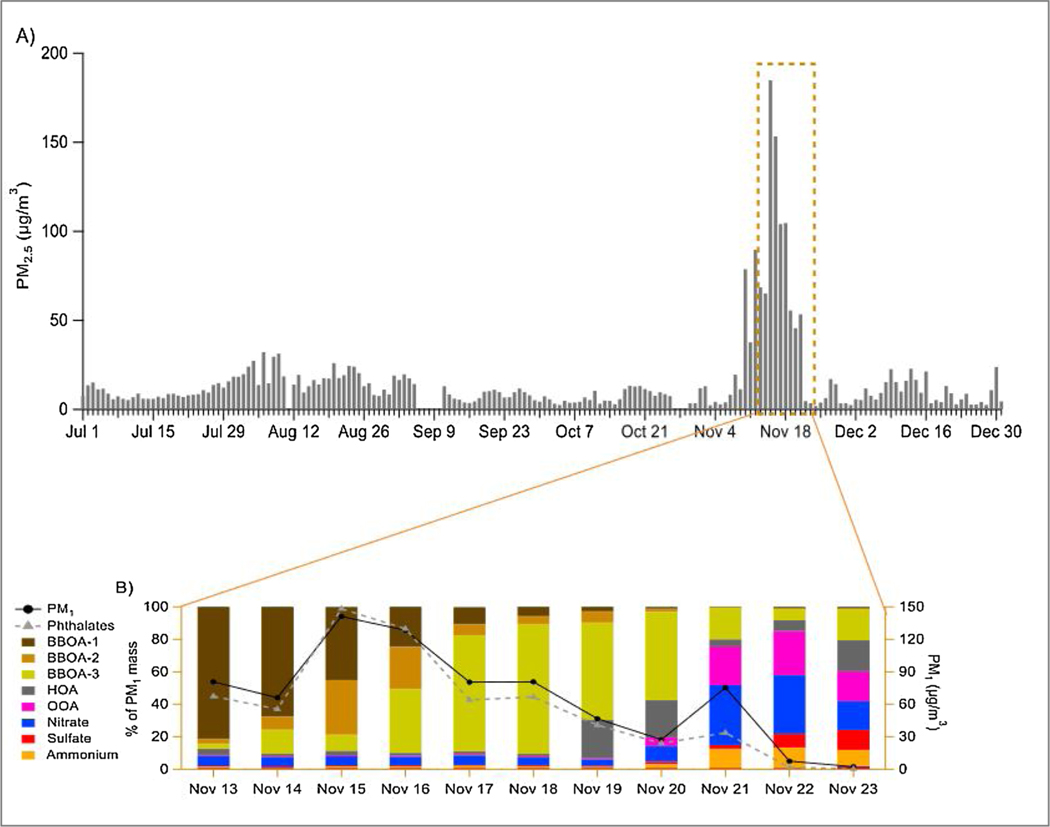
24 -h average concentrations of ambient fine particulate matter (PM_2.5_) measured at the UC Davis site by the California Air Resources Board from July 01 –December 31, 2018. Panel A demonstrates changes in the average concentration and chemical composition of submicrometer particulate matter (PM_1_) during the Fire event. Panel B shows the temporal transition in chemical constituents as percentages of the total PM_1_ mass analyzed by high-resolution time-of-flight aerosol mass spectrometry. Abbreviations: BBOA – biomass burning organic aerosols, with Type 1 representing fresh smoke, Type 2 representing more oxidized/aged smoke than Type 1, and Type 3 representing the most oxidized/aged smoke; HOA – Hydrocarbon-like organic aerosols that are mainly associated with vehicle emissions; OOA – oxidized organic aerosols primarily are formed through atmospheric chemical processes.

Based on measurements made at the UCD site by CARB, the Camp Fire cohort monkeys were exposed to elevated PM2.5 on 12 consecutive days during the 2018–2019 breeding season from July 2018 to December 2018 (Fig. 5). The days corresponded to November 8–20, 2018, the period of the Camp Fire event, when PM2.5 levels exceeded the United States Environmental Protection Agency’s 24 -h National Ambient Air Quality Standard (35 μg/m^3^) and reached levels as high as 185 μg/m3 (Fig. 5).

### 3.3. PM chemical composition

Three types of biomass burning organic aerosols (BBOA-1, BBOA-2, and BBOA-3) were identified by HR-AMS during the Camp Fire period (Fig. 5B). These aerosols differed in their degree of oxidation reflecting varying contributions of fresh (BBOA-1; atomic oxygen to carbon ratio (O/C) =0.58) and aged (BBOA-2 (O/C = 0.64) and BBOA-3 (O/C = 0.78)) smoke particles to the overall PM mass over the course of the fire. Fig. 5A and 5B demonstrate the dominance of wildfire smoke during days of elevated PM from the Camp Fire event with total BBOA (=BBOA-1 +BBOA-2 + BBOA-3) contributing more than 90 % of PM_1_ during the Camp Fire period but less than 20 % after November 21 (Fig. 5B). As the wildfire smoke abated, a transition to a more typical ambient chemical composition of PM was noted, with a low overall PM_2.5_ concentration beginning on 11/20/18 and continuing through 11/23/18 (Fig. 5B, solid line). Though there was a temporary increase in PM_1_ on 11/21, it was driven primarily by the elevation of inorganic aerosol species such as ammonium nitrate. Of additional interest was the distinct finding of a phthalate signal (represented by C_8_H_5_O_3_+ in the AMS spectrum) significantly enhanced in smoke particles during the Camp Fire event (Fig. 5B, dashed line). The phthalate signal was below the detection limit after November 23. A correlation was found between the phthalate signal and BBOA concentration. These results provided evidence that phthalates were enriched in the Camp Fire smoke, likely due to the burning of structures, households, and other anthropogenic objects that contain plastics. Epidemiologic studies suggest that phtha- late exposure is associated with pregnancy loss, infertility, low birth weight, and preterm birth [8].

## 4. Discussion

The elevated levels of PM_2.5_ resulting from the Camp Fire wildfire offered a rare opportunity to study possible effects on pregnancy out- comes in an outdoor colony of primates. Our study demonstrated a correlation between pregnancy loss and exposure to elevated levels of fine particulates (PM_1_ and PM_2.5_) early in gestation. With ongoing drought conditions and wildfire events occurring more frequently than in the past, understanding the effects of wildfire events and resulting air pollution on pregnancy outcomes in non-human primates could provide useful information that may be extrapolated to humans. Although placentation and susceptibility to stress-induced pregnancy loss are important differences, the macaque pregnancy is the closest available animal model for investigating pregnancy loss in humans.

As demonstrated in Fig. 1, most losses occurred in the pregnancies conceived during the month of November (n = 6), when the wildfire occurred. Notably, no change in food or water consumption was detected under the American Association for Laboratory Animal Science (AALAS) approved husbandry management practices. When examining the specific gestational age of the conceptions that went on to miscarry (did not lead to live birth), all exposures occurred within the first 60 days of gestation alluding to a possible window of time during which the pregnancy may be considered most sensitive to the reprotoxic effects of PM_2.5_. This “all or none” theory regarding toxic effects on early pregnancies is similarly demonstrated in human gestations in that embryos exposed to high levels of ionizing radiation, for example, within the first several weeks either miscarry or exhibit no consequence [9]. The impact of radiation is similarly thought to be dose, duration, and gestational-age dependent. Relatedly, increased levels of exposure to carbon monoxide has also been linked to an increased risk of cardiac birth defects with exposures in the second month of human pregnancies. This dose response was demonstrated in a case-control study conducted in Southern California from 1987 to 1993 by evaluating data from the California Birth Defects Monitoring Program [10]. Furthermore, several epidemiological studies have shown associations between elevated levels of air pollution and increased risk of preterm delivery [11] and low birth weight [12].

The present study has several strengths including a real-world exposure of significant intensity and constancy for two weeks which could not have been achieved in a laboratory setting. The availability of numerous non-human primates that were exposed at various time points of conception, implantation, and organogenesis was also fortuitous. While this study was observational and uncontrolled in nature, the long history of the consistent and continuous outdoor housing and routine care during previous years of relatively clean-air breeding seasons, including many of the same animals, provided a rich statistical set of data for analyses. While the exact date of conception was not known, the estimate of gestational stage in early pregnancy by digital palpation and verification of this estimate with a subset of mCG measurements was considered an asset. Weaknesses included the lack of control over the exposure, its duration, consistency, or intensity. In addition, regarding the primate that was reported to not conceive during the 2018–2019 breeding season, there was no way to know whether conception and loss occurred prior to gestational day 14, as mCG is only detectable between day 14–28, and palpation is not a reliable indicator of pregnancy during this early stage. Finally, birth weight was not collected in this study given the inability to physically separate the newborn primates from their mothers immediately after delivery.

Cumulative findings from the present study suggest exposure to elevated wildfire PM_2.5_ levels at a key time, early in pregnancy, may have a negative effect on pregnancy outcomes in the non-human pri- mate. Although the data presented in this study do not definitively demonstrate causation, they demonstrate a correlation and modest trend towards pregnancy loss during the 2018–2019 breeding season (Figs. 2 and 4, Table 3). Similarly, despite the fact the OR and RR were not statistically significant, both point towards a modestly increased risk of pregnancy loss for the wildfire-exposed group. Given the significant physiologic and psychologic investments made in pregnancy outcomes among all species, the ability to reproduce effectively is of paramount importance.

The Camp Fire event destroyed the town of Paradise including homes, vehicles, and all their contents. The presence of plastics and other synthetics associated with these items burning in the fire was evident by the measurement of phthalates in the PM_2.5_ air chemistry of Davis, CA, hundreds of kilometers from the town of Paradise. Other volatile components of the fire were also likely to be present in the smoke plume. Therefore, the adverse effects of the Camp Fire exposure should not be attributed only to biomass products of combustion but also due to the presence of phthalates which have been implicated in adverse reproductive outcomes.

## 5. Conclusions

There is a paucity of data regarding the impact of wildfire smoke exposure on pregnancy outcomes. The present study highlights the possible deleterious effects of wildfire smoke. The results of this study characterize the adverse effects of air pollution on pregnancy outcomes in the non-human primate animal model and, in doing so, suggest that similar effects may be found in human populations. Primates that became pregnant and then went on to lose the pregnancy, did so after exposure to elevated levels of fine particulates. The present study therefore suggests a possible temporal relationship between air pollution exposure, gestational stage, and adverse effects. Further research is needed to evaluate the exact mechanism, dose, and duration in which wildfire smoke affects pregnancy outcomes after exposure early in gestation when a developing embryo may be most sensitive. Given the increase in frequency and severity of wildfires in the setting of climate change, these data may prove to be useful when counseling pregnant individuals regarding the risks of wildfire smoke exposure.

## Figures and Tables

**Table 1:** Pregnancy data of female rhesus macaques from ten consecutive breeding seasons 2009–2019 compared to the 2018–2019 Camp Fire-Exposed Cohort.

Breeding Season Cohort	n	Total Confirmed Conceptions	Conception Rate	Live Births	Live Births / Total Conceptions
2009–2010	911	800	88 %	702	88 %
2010– 2011	759	675	89 %	582	86 %
2011–2012	816	689	84 %	628	91 %
2012–2013	773	671	87 %	621	93 %
2013–2014	718	629	88 %	574	91 %
2014– 2015	803	621	77 %	550	88 %
2015– 2016	549	423	77 %	387	91 %
2016– 2017	541	500	92 %	466	93 %
2017– 2018	798	672	84 %	608	90 %
2018– 2019 Camp Fire-Exposed Cohort	45	45	n/a*	37	82 %

* The group of 45 females that conceived during the Camp Fire period.

**Table 2:** mCG, palpation, and birth outcome data for the cohort of primates in the 2018–2019 breeding season.

Primate Number	Sample Date	mCG (ng/mL)	Pregnant (via initial palpation)	Outcome
37329	4-Dec-18	0.056	+	LV
41403	11-Dec-18	6.875	+	LV
39656	11-Dec-18	2.572	+	LV
39656	11-Dec-18	2.572	+	LV
42740	4-Dec-18	16.152	+	LV
42217	11-Dec-18	15.392	+	LV
43294	4-Dec-18	0.112	+	LV
42048	11-Dec-18	0.267	−	LV
43480	6-Dec-18	0.429	−	D
44041	11-Dec-18	0.428	−	LV
44493	4-Dec-18	0.042	−	LV
42624	4-Dec-18	<0.039	+	LV
43478	4-Dec-18	<0.039	+	LV
43455	4-Dec-18	<0.039	+	LV
45315	11-Dec-18	<0.039	+	D
43815	4-Dec-18	<0.039	+	LV
45142	11-Dec-18	<0.039	+	LV
39761	11-Dec-18	<0.039	+	LV
39573	11-Dec-18	<0.039	+	LV
40329	11-Dec-18	<0.039	+	LV
41938	11-Dec-18	<0.039	+	LV
42606	4-Dec-18	<0.039	+	LV
43731	4-Dec-18	<0.039	+	LV
44344	4-Dec-18	<0.039	+	LV
40567	11-Dec-18	<0.039	+	LV
42552	6-Dec-18	<0.039	+	D
44122	11-Dec-18	<0.039	+	LV
44197	11-Dec-18	<0.039	+	LV
44366	11-Dec-18	<0.039	+	LV
44815	11-Dec-18	<0.039	+	LV
45180	11-Dec-18	<0.039	+	LV
41273	11-Dec-18	<0.039	+	LV
42327	4-Dec-18	<0.039	+	LV
43491	11-Dec-18	<0.039	+	LV
44541	11-Dec-18	<0.039	+	LV
44571	6-Dec-18	<0.039	+	D
45093	11-Dec-18	<0.039	+	D
41972	11-Dec-18	<0.039	+	LV
43871	11-Dec-18	<0.039	+	LV
39655	11-Dec-18	<0.039	+	LV
42079	11-Dec-18	<0.039	+	D
44449	4-Dec-18	<0.039	+	LV
43764	11-Dec-18	<0.039	+	LV
44566	4-Dec-18	<0.039	+	LV
40103	11-Dec-18	<0.039	−	D
38658	6-Dec-18	<0.039	−	LV
40256	6-Dec-18	<0.039	−	LV
40278	6-Dec-18	<0.039	−	LV
41004	11-Dec-18	<0.039	−	LV
41624	11-Dec-18	<0.039	−	LV
41782	11-Dec-18	<0.039	−	LV
41804	11-Dec-18	<0.039	−	LV
42022	4-Dec-18	<0.039	−	LV
42098	6-Dec-18	<0.039	−	LV
42309	4-Dec-18	<0.039	−	LV
42721	4-Dec-18	<0.039	−	LV
43310	11-Dec-18	<0.039	−	LV
43632	6-Dec-18	<0.039	−	D
43805	6-Dec-18	<0.039	−	LV
44061	11-Dec-18	<0.039	−	LV
44390	6-Dec-18	<0.039	−	LV
44536	4-Dec-18	<0.039	−	LV
44595	4-Dec-18	<0.039	−	LV
44609	11-Dec-18	<0.039	−	LV
44832	11-Dec-18	<0.039	−	NC
45340	6-Dec-18	<0.039	−	LV

Abbreviations: + (plus = pregnant; - (minus = not pregnant; LV = Live vaginal birth, D = Demised fetus after confirmation of pregnancy, NC = No Conception.

**Table 3:** Conception outcome data of female rhesus macaques exposed to the Camp Fire smoke “Camp Fire-Exposed Cohort” compared to control of 9 prior breeding seasons (2009 – 2018).

	Pregnancy Loss (% of Total)	Live Birth (% of Total)	Total
Camp Fire-Exposed Cohort	8 (18 %)	37 (82 %)	45
Control	562 (10 %)	5118(90 %)	5680

## References

[1] Formenti P, Elbert W, Maenhaut W, Haywood J, Osborne S, Andreae MO, Inorganic and carbonaceous aerosols during the Southern African Regional Science Initiative (SAFARI 2000) experiment: chemical characteristics, physical properties, and emission data for smoke from African biomass burning, Am. J. Geogr. Res. Rev. 108 (2003) 8488, 10.1029/2002JD002408,D13.

[2] Cohen AJ, Bauer M, Burnett R, Anderson HR, Frostad J, Estimates and 25-year trends of the global burden of disease attributable to ambient air pollution: an analysis of data from the Global Burden of Diseases Study 2015, Lancet 389 (2017) 1907–1918, 10.1016/S0140-6736(17)30505-6. 28408086 PMC5439030

[3] Holstius DM, Reid CE, Jesdale BM, Morello-Frosch R, Birth weight following pregnancy during the 2003 Southern California wildfires, Environ. Health Perspect. 120 (2012) (2012) 1340–1345, 10.1289/ehp.1104515, 9. 22645279 PMC3440113

[4] Lamichhane DK, Leem JH, Lee JY, Kim HC, A meta-analysis of exposure to particulate matter and adverse birth outcomes, Environ. Health Toxicol. 30 (2015), e2015011, 10.5620/eht.e2015011. PMC4722965 26796890

[5] Silk J, Short J, Roberts J, Kusnitz J, Int. J. Primatol. 14 (1993) 95–104, 10.1007/BF02196505.

[6] Lohstroh P, Laughlin L, Gee N, Lasley B, Development, validation and application of a chemiluminescent immunoassay for the measurement of circulating chorionic gonadotropin levels in the laboratory macaque, J. Med. Primatol. 36 (3) (2007) 164–169, 10.1111/j.1600-0684.2006.00189. 17517091

[7] Canagaratna M, Jayne J, Jimenez JL, Allan JA, Alfarra R, Zhang Q, Onasch T, Drewnick F, Coe H, Middlebrook A, Delia A, Williams L, Trimborn A, Northway M, DeCarlo P, Kolb C, Davidovits P, Worsnop D, Chemical and microphysical characterization of ambient aerosols with the aerodyne aerosol mass spectrometer, Mass Spectrom. Rev. 26 (2007) 185–222. DOI:10.1002/mas.20115, 2007. 17230437

[8] Kay VR, Chambers C, Foster WG, Reproductive and developmental effects of phthalate diesters in females, Crit. Rev. Toxicol. 43 (3) (2013) 200–219, 10.3109/10408444.2013.766149. 23405971 PMC3604737

[9] De Santis M, Cesari E, Nobili E, Straface G, Cavaliere AF, Caruso A, Radiation effects on development, Birth Def. Res. Part C Embryo Today 81 (3) (2007) 177–182, 10.1002/bdrc.20099,2007. 17963274

[10] Ritz F. Yu, S. Fruin, G. Chapa, G. Shaw, J. Harris, Ambient air pollution and risk of birth defects in Southern California, Am. J. Epidemiol. 155 (1) (2002) 17–25, 10.1093/aje/155.1.17. 11772780

[11] Xu X, Ding H, Wang X, Acute effects of total suspended particles and sulfur dioxides on preterm delivery: a community-based cohort study, Arch. Environ. Occup. Health 50 (6) (1995) 407–415, 10.1080/00039896.1995.9935976. 8572718

[12] Wang X, Ding H, Ryan L, Xu X, Association between air pollution and low birth weight: a community-based study, Environ. Health Perspect. 105 (5) (1997) 514–520, 10.1289/ehp.97105514. 9222137 PMC1469882

